# Cardiac Valvular Inflammatory Pseudotumor

**DOI:** 10.1186/1749-8090-3-53

**Published:** 2008-09-29

**Authors:** Pradeep Vaideeswar, Anil M Patwardhan, Pragati A Sathe

**Affiliations:** 1 Department of Pathology (Cardiovascular & Thoracic Division), Seth G. S. Medical College and K. E. M. Hospital, Mumbai, India; 2 Dr. P. K. Sen department of Cardiovascular & Thoracic Surgery, Seth G. S. Medical College and K. E. M. Hospital, Mumbai, India

## Abstract

Inflammatory pseudotumors are quasineoplastic lesions that occur in the lungs as well as other extrapulmonary sites. The heart is an uncommon site of origin. We report a valvular pseudotumor that produced chronic mitral and aortic regurgitation in an elderly woman.

## Introduction

Diseases of the heart valves cut across all age groups, race and geographic locations to form a significant cause of morbidity and mortality. These deformities may be congenital or acquired, as a result of developmental, post-inflammatory and degenerative changes. In our country, owing to high prevalence of rheumatic heart disease [1], the etiology in most valvular dysfunctions (stenosis and/or regurgitation) is attributed to rheumatic heart disease, unless proved otherwise. This is largely true in most instances, but there do exist occasions, where a "rheumatic "assumption is erroneous. We present a rare case of inflammatory pseudotumor, manifesting as a granulomatous valvulitis, producing chronic mitral and aortic regurgitation in a 62 years old lady.

## Case report

A 62-year-old woman presented to our Cardiovascular & Thoracic Center with complaints of breathlessness and palpitation. The dyspnea (grade II), present for the past eight years, had progressed in six months to grades III/IV. She had had past hospital admissions for similar complaints. A secundum atrial septal defect had been closed in the year 1990. She was advised valve replacement for rheumatic valvular heart disease. Routine hematological and biochemical investigations had been normal. Two-dimensional echocardiography showed severe mitral and moderate aortic, regurgitation. The pulmonary arterial pressure was 80 mm Hg. She was taken up for mitral and aortic valve replacements. The anterior mitral leaflet was thick and fleshy while the posterior leaflet was thin. There was no significant sub-valvular pathology. The anterior leaflet and the medial scallop of the posterior leaflet were excised and the valve was replaced by Carbomedics mechanical valve (27 mm). The right and non-coronary cusps of the aortic valve showed similar thickening. The cusps were excised and the valve replaced by a St. Jude's mechanical valve (19 mm). During surgery, there was inadvertent tear of the superior caval vein, which was sutured and the patient was put on ventilator support. She expired eight hours after surgery.

**Figure 1:**
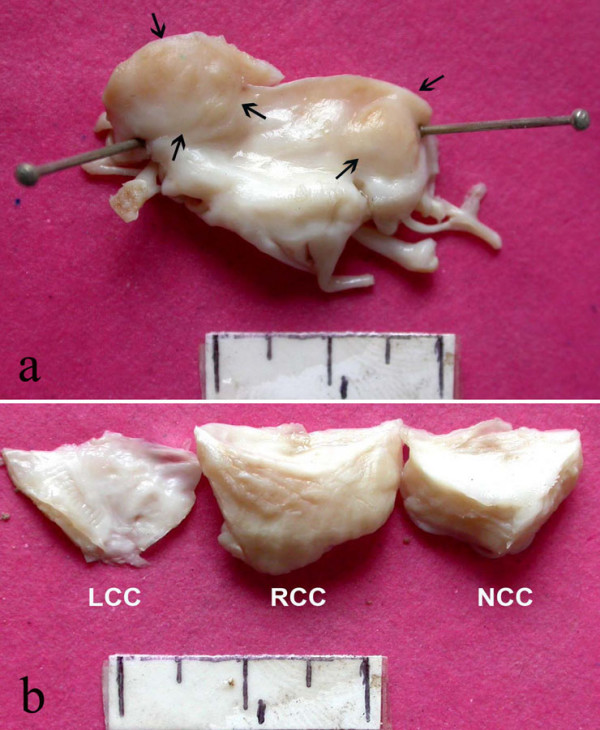
**a) Excised anterior mitral leaflet showing nodularity (arrows) at its cut margins.** b) Uniform, marked thickening of the right and non-coronary cusps of the aortic valve. Note mild thickening of the left cusp.

**Figure 2:**
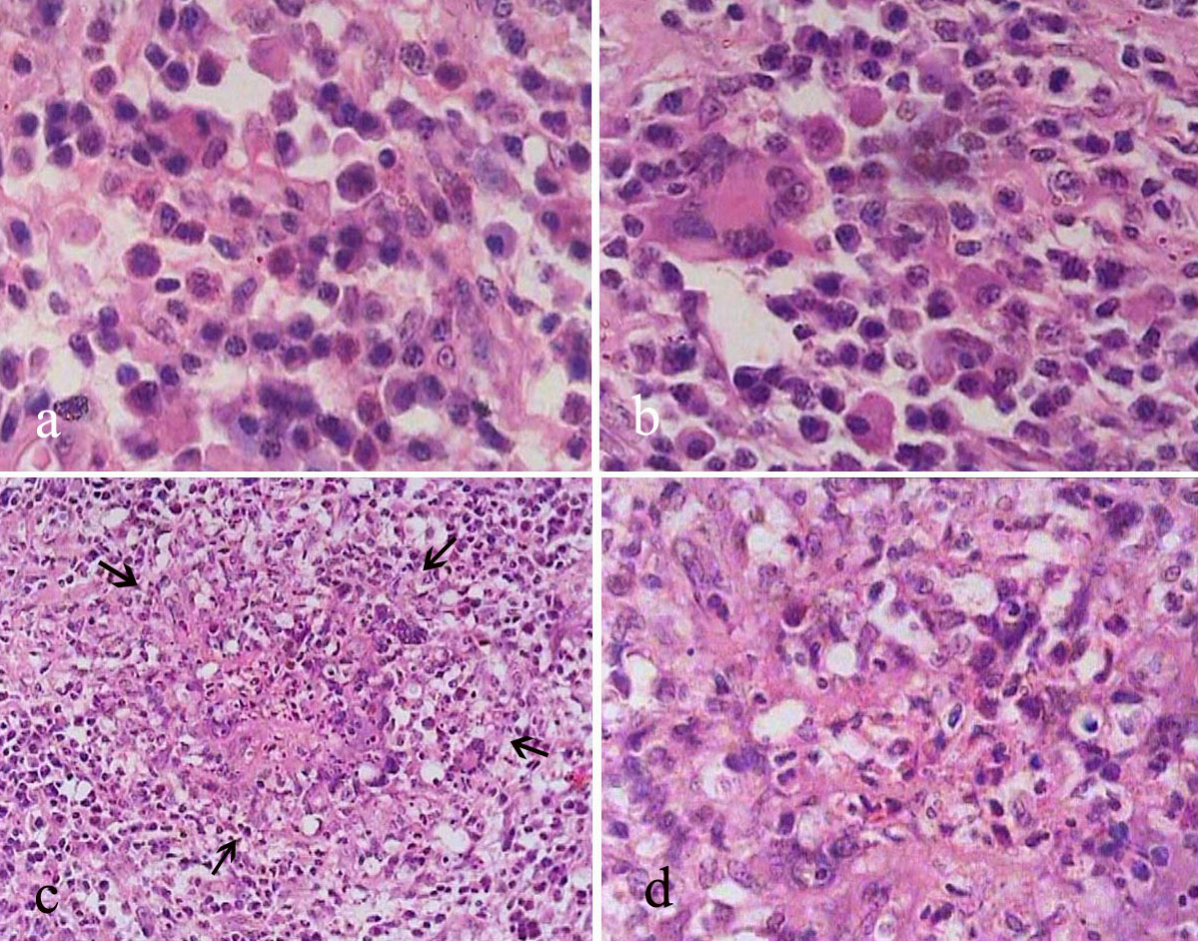
a. Infiltrate of lymphocytes, plasma cells and histiocytes, b. Presence of giant cells (H&E × 400), c. A vaguely palisaded granulomatous reaction (arrows, H&E, × 250), d. Higher magnification to show neutrophils and nuclear debris (H&E × 400).

On gross examination, the anterior leaflet (Figure 1), at its basal, commissural aspects was nodular and firm. The remaining leaflet was mildly thickened with rolling of the free margin. The tendinous chords were discrete, but moderately thickened. The right and non-coronary cusps were markedly thickened and firm; the left cusp looked normal (Figure 1). The thickened portions of both valves had a homogeneous, creamy cut surface. No thrombi were identified on gross. On histology, these valves revealed extensive polymorphic inflammatory infiltrate in their entire thickness with associated vascularization and minimal collagenization. There were plasma cells, lymphocytes, histiocytes and clusters of multinucleated giant cells (Figure 2), with few eosinophils and neutrophils. Interestingly, there were total seven foci of central ill-defined necroses, accompanied by nuclear debris and neutrophils (Figure 2); these were surrounded by a vague palisade of histiocytes and giant cells. Stains for microorganisms, including fungi and acid fast bacilli, were negative. Since no obvious vegetations were formed, we entertained a possibility of Q fever endocarditis, but the organism (*Coxiella burnetii*) could not be demonstrated on immunohistochemistry or in-situ hydridization. We designated this lesion initially as idiopathic granulomatous valvulitis, but on the basis of exclusion, a diagnosis of valvular inflammatory pseudotumor was finally made. Immunohistochemistry confirmed the polyclonality of the inflammatory cells; myofibroblastic cells were not identified. In the partial chest autopsy that was subsequently performed, similar inflammatory cells were identified at the anterior mitral annulus and in the walls of the right and non-coronary sinuses of Valsalva. There was no involvement of the preserved posterior mitral leaflet or the ascending aorta.

## Discussion

Our patient, a woman in her early sixties, had long-standing mitral and aortic regurgitation. She had had secundum atrial septal defect, patched at the age of 45 years. This type of defect is known to coexist with mitral valvular disease, especially rheumatic [2]. However, review of the valvular morphology at operation and the subsequent valvectomy, did not suggest a rheumatic etiology. More interesting was the presence of inflammation that produced a tumorous expansion of the leaflet/cusps or an "inflammatory pseudotumor". Such inflammatory pseudotumors (IPT) or inflammatory myofibroblastic tumors (IMT), apart from the lungs, are also described in other organs, but occurrence in the heart is rare. Since its first description in 1975 [3], fewer than 30 cases of cardiac IMT have been described [4,5]. By and large, the tumors have manifested mainly in children as inflow or outflow obstructions due to a propensity to involve the chambers [5]. The present case is the eleventh case to be reported in adults [5-7]. Apart from cardiac symptoms, our patient did not have any other constitutional symptoms, seen with IPTs [5].

The IMT are considered as quasineoplastic lesions composed of inflammatory cells and myofibroblasts. In one subset of IMT, there is a profusion of inflammatory cells, secondary to infective or non-infective immune reactions, while the other group (considered neoplastic) is composed of myofibroblastic proliferation [8]. We saw a predominance of inflammatory cells, including multinucleated giant cells in the excised mitral and aortic valves; the entire tissue had been processed. Hence we have preferred to use the term IPT. So far only nine patients have had involvement of the atrioventricular or the arterial valves with or without concomitant chamber involvement [5-7,9-11]. It is interesting to note that lesions which tended to infiltrate the entire thickness of the valve leaflet/cusp were predominantly inflammatory [6,7,9-11] while those having a polypoidal configuration were rich in spindled cells [5].

No organisms were identified by special stains performed for bacteria, Mycobacteria and fungi. Assuming that there was a subtle underlying mitral valvulopathy due to the defect in the inter-atrial septum, we thought that a strong contender for producing valvulitis is chronic Q fever, where endocarditis accounts for 60 to 70% of the cases [12]. The patients are often afebrile, and interestingly, vegetations are absent or small [12]. However, *Coxiella burnetii *was not detected. One must also bear in mind other rarer causes of granulomatous valvulitis such as rheumatoid arthritis [13], Wegener's granulomatosis [14], and large vessel vasculitides [15]. Our patient did not have any manifestations, suggestive of the aforementioned diseases.

Taking into consideration the rarity of cardiac IMT, the prognosis and optimal therapeutic measure to be adopted is uncertain. In most instances, partial or complete resection is advocated as some of the lesions can be potentially fatal depending on their location [5]. There has been a report where corticosteroid therapy had produced marked regression in size of the lesion [7]. However, long-term follow-up are not available in many cases.

## Competing interests

The authors declare that they have no competing interests.

## Authors' contributions

PV participated in the design of the study and review of literature. AMP was responsible for the final editing of the manuscript. PAS participated in the review of literature. All authors read and approved the final manuscript.
